# *Thymus vulgaris* Essential Oil Protects Zebrafish against Cognitive Dysfunction by Regulating Cholinergic and Antioxidants Systems

**DOI:** 10.3390/antiox9111083

**Published:** 2020-11-04

**Authors:** Luminita Capatina, Elena Todirascu-Ciornea, Edoardo Marco Napoli, Giuseppe Ruberto, Lucian Hritcu, Gabriela Dumitru

**Affiliations:** 1 Department of Biology, Faculty of Biology, Alexandru Ioan Cuza University of Iasi, 700506 Iasi, Romania; luminita.capatina@student.uaic.ro (L.C.); ciornea@uaic.ro (E.T.-C.); gabriela.dumitru@uaic.ro (G.D.); 2 Institute of Biomolecular Chemistry, National Research Council ICB-CNR, 95126 Catania, Italy; edoardo.napoli@icb.cnr.it (E.M.N.); giuseppe.ruberto@icb.cnr.it (G.R.)

**Keywords:** *Thymus vulgaris*, essential oil, scopolamine, anxiety, memory, oxidative stress

## Abstract

*Thymus vulgaris* L. is an aromatic herb used for medicinal purposes such as antimicrobial, spasmolytic, antioxidant, anti-inflammatory, antinociceptive, antitumor, and may have beneficial effects in the treatment of Alzheimer’s disease. The present study aimed to investigate whether *Thymus vulgaris* L. essential oil enhances cognitive function via the action on cholinergic neurons using scopolamine (Sco)-induced zebrafish (*Danio rerio*) model of memory impairments. *Thymus vulgaris* L. essential oil (TEO, 25, 150, and 300 µL/L) was administered by immersion to zebrafish once daily for 13 days, whereas memory impairment was induced by Sco (100 μM), a muscarinic receptor antagonist, delivered 30 min before behavioral tests. Spatial memory was assessed using the Y-maze test and novel object recognition test (NOR). Anxiety and depression were measured in the novel tank diving test (NTT). Gas Chromatograph-Mass Spectrometry (GC-MS) analysis was used to study the phytochemical composition of TEO. Acetylcholinesterase (AChE) activity and oxidative stress response in the brain of zebrafish were determined. TEO ameliorated Sco-induced increasing of AChE activity, amnesia, anxiety, and reduced the brain antioxidant capacity. These results suggest that TEO may have preventive and/or therapeutic potentials in the management of memory deficits and brain oxidative stress in zebrafish with amnesia.

## 1. Introduction

Alzheimer’s disease (AD) is a progressive neurodegenerative condition with significant variability in clinical characteristics and biomarkers and numerous genetic and environmental factors implicated in its etiology and development [1,2]. Moreover, AD is the most prevalent type of dementia in the world and is medically characterized as the pathological deposition of folded β-amyloid (Aβ) plaques and hyperphosphorylated neurofibrillary tau tangles in the brain leading to neurodegeneration [3,4]. Clinically, AD poses issues of progressive episodic memory and executive performance across cognitive continuums ranging from cognitively unimpaired (CU), moderate cognitive impairment (MCI), to AD [3]. Numerous studies on the brains of AD and dementia patients have consistently reported defects or damage to central cholinergic pathways [5]. Given this, the treatment of AD-related dementia is typically done through the use of acetylcholinesterase (AChE) inhibitors, such as donepezil [6]. Non-cognitive symptoms associated with AD and related dementias include insanity (delusions, hallucinations), mood disorders (depression, euphoria, irritability, anxiety), behavioral changes (disinhibition, apathy), agitation, anger, slowness, wandering, altered sexual activity, modified sleep habits, and eating disorders [7]. Oxidative stress is known to be a very significant factor in ageing and age-related disorders and a large amount of research has shown that oxidative stress is an important pathogenic factor in AD [8]. Protein oxidation markers, such as protein carbonyls, have been shown to be increased in AD brains in areas with proven histopathological AD characteristics [9]. Protein carbonyls and 3-nitrotyrosine (3-NT) levels have been increased in the frontal cortex of individuals with MCI, mild AD, and AD, with no distinction between disease stages, a finding that supports the concept of oxidative stress as an early event in AD [10].

Scopolamine (Sco) is a cholinergic antagonist of the muscarinic acetylcholine receptors known to interfere with acetylcholine production in the central nervous system. This drug was used as a guide for the induction of amnesia in animal models [11,12,13,14]. The most important characteristic of dementia is memory deterioration, which may be chemically caused by the use of Sco in laboratory animals. The effect of Sco on memory is followed by central cholinergic dysfunction and oxidative stress in zebrafish [15]. Sco has been characterized as an amnestic agent in zebrafish and is widely used to study memory formation and rescue in conjunction with nootropic and memory-enhancing drugs [12]. Additionally, Sco has been reported to induce cholinergic deficits and memory impairment via the up-regulation of AChE activity [16]. Furthermore, there is increasing evidence that Sco interferes with the molecular homeostasis of the extracellular signal-regulated kinase (ERK) and cAMP response element-binding (CREB)/brain-derived neurotrophic factor (BDNF) in animal models [17,18] while simulating ERK/CREB/BDNF injury in the brains of patients with AD [19,20].

There is no treatment for AD. However, there are medications available that can alleviate symptoms temporarily. Currently, only a few medications are available on the market with multiple adverse effects. Donepezil is the second approved AChE inhibitor for the treatment of mild to moderate AD by the United States Food and Drug Administration (FDA), extensively used worldwide [21]. As donepezil is generally tolerated, most adverse events are nausea, vomiting, diarrhea, muscle cramps, fatigue, and weight loss [22]. Additionally, rivastigmine commonly used as an AChE inhibitor could induce side effects such as nausea, vomiting, diarrhea, weight loss, indigestion, and muscle weakness [23]. Besides, memantine used for treating dementia exhibited different side effects such as dizziness, headache, diarrhea, constipation, and confusion [24]. Natural products from plants represent interesting candidates for the treatment of AD and the present study aimed to identify if the *Thymus vulgaris* essential oil (TEO) is a potent source of bioactive compounds to counteract AD [25].

The genus *Thymus* (*Lamiaceae* family) comprises over 300 species of aromatic perennial herbaceous plants with important medicinal properties [26]. Thyme oil has been widely used in the food, pharmaceutical, and cosmetic industries due to its various biological processes. Biological properties include antioxidant, anti-inflammatory, anti-tumor, and anti-microbial effects [27].

The chemical composition of the TEO has been documented. Thymol has generally been identified to be the main constituent of *Thymus vulgaris*, accompanied by carvacrol and linalool [28]. These compounds have been stated to have also therapeutic properties (e.g., vs. AD) [29]. For instance, Asadbegi et al. [30] demonstrated that thymol decreased the effect on memory damage caused by intrahippocampal injection of the Aβ peptide in high-fat diet-fed rats and could be found as neuroprotective. Additionally, Capibaride et al. [31], confirmed the marked antidepressant-like effect of thymol, as demonstrated by its ability to reverse chronic corticosterone-associated behavior and a decrease in BDNF levels in female mice. Furthermore, Formiga et al. [32] suggested the intestinal anti-inflammatory function of *p*-cymene and rosmarinic acid involving the cytoprotection of the intestinal barrier, the preservation of the mucus layer, and the conservation of communicating junctions, as well as the regulation of the antioxidant and immunomodulatory processes. Azizi et al. [33] demonstrated that the neuroprotective effects of carvacrol and thymol against Aβ25–35 might be through attenuating oxidative damage and increasing the activity of PKC as a memory-related protein. Besides, β-myrcene and caryophyllene within the monoterpenes and sesquiterpenes have been identified as the major compounds. β-myrcene is known to have analgesic, anxiolytic, and anti-inflammatory effects [34,35]. Caryophyllene was known as an anti-inflammatory compound in some *Cannabis* extracts due to interactions with cannabinoid receptors and gastric cytoprotective activity [36]. Notably, caryophyllene oxide appears to be a multi-target molecule known for its anti-cancer and analgesic properties [35].

In zebrafish, the essential oils from *Thymus quinquecostatus* Celak. from the Loess Plateau in China exhibited antioxidant potential mainly by regulating the Nrf2/Keap1 signaling pathway. Rabiei et al. [37] reported that *Thymus vulgaris* extract has remedial on memory and behavioral disturbances caused by Sco and may have beneficial effects in the treatment of AD. Elaissi et al. [38] demonstrated antiproliferative activities of *Thymus numidicus* Poir. against two human cancer cell lines: the colonic (HCT116) and breast adenocarcinoma (MCF7), respectively. Butt et al. [39] demonstrated that the TEO was found to be a good source of thymoquinone as a chemotherapeutic drug that expressed potent antioxidant and antiproliferative activities. To date, no study indicated the TEO positive effects on memory function in zebrafish against Sco-induced cognitive dysfunction by regulating cholinergic and antioxidant pathways. The present study was therefore organized to examine the phytochemical composition of the TEO and to evaluate the effects on anxiety, cognitive performance, and the brain antioxidant capability in a zebrafish Sco model of amnesia.

## 2. Materials and Methods

### 2.1. Essential Oil and Chemical Material

The TEO used in this study is a commercial sample produced with organic plant material and kindly supplied by Flora S.R.L. (Lorenzana, Pisa, Italy), batch no. 162371. The standard mix of *n*-alkanes C_9_–C_22_ was purchased by Alltech (Bologna, Italy).

### 2.2. Gas Chromatograph-Mass Spectrometry (GC-MS) Analysis

To obtain the chromatographic profile of the essential oil a Shimadzu GC-17A Gas Chromatograph (Shimadzu, Milan, Italy) equipped with a 15 m × 0.1 mm × 0.1 mm fused silica capillary column (Supelco SPB^TM^-5, Merk KGaA, Darmstadt, Germany) and Flame Ionization Detector (FID) was used. GC-MS analyses were performed on a Shimadzu GCMS-QP5050A (Shimadzu, Milan, Italy). The operating conditions for both runs were the following: 60 °C for 1 min, 60–280 °C at 10 °C/min then 280 °C for 1 min; injector temperature 250 °C; detector temperature 280 °C; carrier gas helium (1 mL/min); split mode (1:200), the volume of injection 1 mL (4% essential oil/CH_2_Cl_2_ *v*/*v*). Percentages of compounds were determined from their peak areas in the GC-FID profiles. Mass spectrometer parameters were the following: Ionization at 70 eV, ion source temperature 180 °C. Mass spectral data were acquired in the scan mode in m/z range 40–400. Oil solutions were injected with the split mode (1:96) [40].

The identity of components was based on their retention index relative to C_9_–C_22_ *n*-alkanes on the SPB^TM^-5 column and computer matching of spectral MS data with those from NIST MS 107 and NIST 21 libraries [41], the comparison of the fragmentation patterns with those reported in the literature [42].

### 2.3. Animals

**Figure 1:**
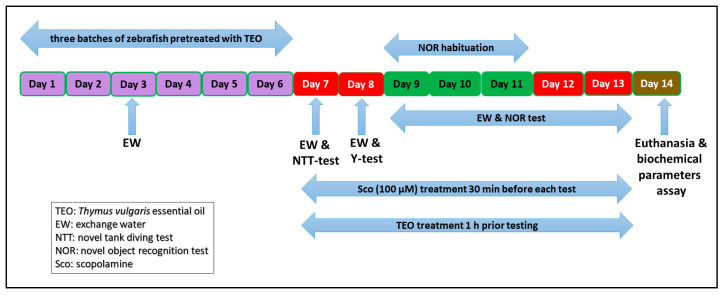
Experimental design procedure for the *Thymus vulgaris* essential oil administration, behavioral study, and biochemical analysis.

50 adults zebrafish (*Danio rerio*) wild-type short-fin strain of both sexes (ratio: 50:50 male: Female, 3–4 month-old, and 3–4 cm-long) were purchased from a specialized commercial supplier (Pet Product S.R.L., Bucharest, Romania). The zebrafish were acclimatized in the experimental room for at least 14 days. Fish were sorted in groups of 10 fish in 24 L thermostated (26 ± 1 °C) tanks, kept under water filtration and aeration (7.2 mg O_2_/L) using Tetra*tec*^®^ air pumps (Tetra, Melle, Germany). Animals were maintained on 14/10 h light/dark cycle, and were fed twice a day with Norwin Norvitall flake (Norwin, Gadstrup, Denmark). In behavioral studies, acclimatized zebrafish were randomly assigned to the control, the Sco (100 µM), and three TEO treatment groups (25, 150, and 300 µL/L). The essential oil [43,44] and the Sco [45] doses were selected according to previous studies. TEO (25, 150, and 300 µL/L) was diluted with 1% Tween-80 solution and was administered to zebrafish by immersion for 1 h, once daily for 13 days, while Sco (100 µM) was administered 30 min before each behavioral tests. The control group was immersed only in unchlorinated water with a 1% Tween-80 solution. All experiments (as outlined in Figure 1) were carried out following scrutiny by the Ethics Committee on Animal Research of the Alexandru Ioan Cuza University of Iasi, Faculty of Biology (Iasi, Romania) under license no. 02/30.06.2020 and fully complied with the Directive 2010/63/EU of the European Parliament and of the Council of 22 September 2010 on the safety of animals. The health status and the well-being of all animals involved in the research have been tested regularly during the behavioral tests. No procedures have caused serious pain or long-lasting damage to the zebrafish, and no experimental subject has died during the experimental procedures (fish housing and behavioral tests).

### 2.4. Behavioral Assay

#### 2.4.1. Novel Tank Diving Test (NTT)

The NTT is a particular test used to evaluate anxiety in zebrafish, as defined by Cachat et al. [46]. The trapezoidal tank (1.5 L) used measured 15.2 height × 27.9 top × 22.5 bottom × 7.1 width cm, equally divided into two horizontal sections (top and bottom). Following 1 h of TEO treatment, the animals were placed individually within the test tank without acclimatization, and swimming behavior was recorded for 6 min. The animals were recorded with a Logitech HD Webcam C922 Pro Stream camera (Logitech, Lausanne, Switzerland) placed 30 cm away from the tank and the videos were analyzed using ANY-Maze^®^ software (Stoelting CO, Wood Dale, IL, USA). Representative tracking images of the zebrafish locomotor activity for each group was obtained at the end of the analysis with ANY-Maze^®^ software. The following parameters heve been recorded: The time spent in the top/bottom zone of the tank (s), total distance traveled (m), and average velocity (m/s).

#### 2.4.2. Y-Maze Test

Spatial memory and the response to novelty in zebrafish was assessed using the Y-maze task [47]. The location in the Y-maze task was considered to be a memory index [45]. The apparatus consisted in a Y-maze glass tank with three identical arms (25 cm long, 8 cm wide, and 15 cm high), filled with 3 L of the the home aquarium water. The water high in the Y-maze was 13 cm. Explicit geometric shapes (squares, circles, and triangles) were placed on the outer walls and visible from the inside. The Y-maze test consisted in two trials separated by 1 h interval. During the first trial, 1 h after TEO treatment, the fish could freely swim in the start arm and in the other arm for 5 min while the novel arm was blocked by a dividing wall. In the second trial, the wall was removed and the fish could explore for 5 min all three arms including the novel environment constituted by the novel arm. Fish were placed in different arms as starting points and the maze was rotated in each experiment to randomize the maze cues. The water was changed between groups and trials. The behavior was fully analyzed using the ANY-Maze^®^ software (Stoelting CO, Wood Dale, IL, USA), assessing time spent in each arm (percent of the total time), total distance traveled (m), turn angle (°), and the preference percentages. The preference percentages were calculated as (time of exploration of the novel arm/time of exploration of the start + time of exploration of the other arm + time of exploration of the novel arm × 100).

#### 2.4.3. Novel Object Recognition Test (NOR)

NOR is a widely used behavioral assay in zebrafish to examine the memory efficiency [48]. The experimental apparatus consists of a 20 L glass tank (30 × 30 × 30 cm) filled with 6 cm of water. The NOR test consists of three phases. In the habituation phase, each animal explores the tank in the absence of the objects for 5 min twice a day (5 h interval between habituation sessions) over 3 consecutive days. In the training phase (on the 4th day), the animals were exposed to two identical hard plastic red cubes for 10 min. In the test phase (1 h after the training phase), a novel object (N, green cube) replaced one of the copies of the familiar objects (F, red cubes), and the exploration time of each object was evaluated for 10 min. The exploration area was established by increasing once the size of the object area; thus, we considered exploration when the fish were up to 2.5 cm far from each side of the object. The behavior was fully analyzed using the ANY-Maze^®^ software (Stoelting CO, Wood Dale, IL, USA), assessing the exploratory time (s) and the preference percentages. The preference percentages have been determined as (time of exploration of N/time of exploration of F + time of exploration of N × 100) [15].

### 2.5. Biochemical Parameters Assay

Immediately after behavioral tests, all zebrafish were euthanized (10 min immersion in ice-water, 2–4 °C) until loss of opercular motions [49], and then, the brains were isolated for the analysis of the biochemical parameters. In ice 0.1 M potassium phosphate buffer (pH 7.4), 1.15% KCl with Potter Homogenizer, the brains were gently homogenized. The resulted homogenate was centrifuged at 960× *g* for 15 min. The supernatant was used for the estimation of acetylcholinesterase (AChE), superoxide dismutase (SOD), and glutathione peroxidase (GPX) specific activities, the total content of reduced glutathione (GSH), protein carbonyl, and malondialdehyde (MDA) level.

#### 2.5.1. AChE Activity

The activity of acetylcholinesterase (AChE, EC 3.1.1.7) was determined by the defined approach as previously described by Ellman et al. [50], using acetylthiocholine chloride (ATC) as artificial substrate. The reaction mixture (600 μL) included 0.26 M phosphate buffer with pH 7.4, 1 mM 5.5′-dithio-bis-2 nitrobenzoic acid (DTNB), and 5 mM ATC. The assay was started by adding supernatant and then by developing the yellow color at 412 nm for 10 min at room temperature. Suitable controls have been performed for the non-enzymatic hydrolysis of ATC. The specific activity of the enzyme was formulated as nmol of ACT/min per/mg of protein.

#### 2.5.2. SOD Activity

The activity of superoxide dismutase (SOD, EC 1.15.1.1) was determined as described by Winterbourn et al. [51]. The reaction mixture (1.5 mL) contained 100 mM TRIS/HCl (pH 7.8), 75 mM NBT, 2 μM riboflavin, 6 mM EDTA, and 200 μL supernatant. The monitoring of the increase in absorbance at 560 nm followed the blue formazan output. One unit of SOD is defined as the amount of the enzyme required to inhibit the rate of NBT reduction by 50%. The specific activity of the enzyme was expressed in units/mg protein.

#### 2.5.3. GPX Activity

Glutathione peroxidase (GPX, E.C. 1.11.1.9) activity was analyzed as described by Sharma and Gupta [52]. A reaction mixture consisting of 1 mL 0.4 mM phosphate buffer (pH 7.0) containing 0.4 mM EDTA, 1 mL of 5 mM NaN_3_, 1 mL of 4 mM glutathione (GSH), and 200 μL of supernatant was pre-incubated at 37 °C for 5 min. Then 1 mL of 4 mM H_2_O_2_ was inserted and incubated at 37 °C for a further 5 min. The GSH excess was quantified by the 5,5′-dithiobis-2-nitrobenzoic acid (DTNB) method. One unit of GPX is defined as the amount of enzyme required to oxidize for 1 nmol GSH/min. The enzyme specific activity was expressed as units/mg protein.

#### 2.5.4. GSH Content

The reduced glutathione (GSH) content was assessed in the zebrafish brain supernatant using the method of Fukuzawa and Tokumura [53]. 200 µL of brain supernatant was mixed with 1.1 mL of 0.25 M sodium phosphate buffer (pH = 7.4) followed by the addition of 130 µL DTNB 0.04%. Finally, the mixture was fixed to a final volume of 1.5 mL with distilled water, and absorbance was read at 412 nm using a spectrophotometer. The results were presented as µg GSH/µg protein.

#### 2.5.5. Protein Carbonyl Level

By measuring the content of protein carbonyl groups, the degree of protein oxidation in the brain was evaluated using a method defined by Oliver et al. [54], and updated by Luo and Wehr [55]. The supernatant fraction was divided into two equal aliquots, each containing around 2 mg of protein. Using 10% trichloroacetic acid (TCA, *w*/*v*, final concentration), both aliquots were precipitated. Another sample was treated with 2 N HCl, and another sample was treated with an equal volume of 0.2% (*w*/*v*) DNPH in 2 N HCl. At 25 °C, both samples were incubated and then stirred at intervals of 5 min. The results were expressed as nmol/mg protein.

#### 2.5.6. MDA Level

A malondialdehyde (MDA) level assay was performed according to previous protocol described by Ohkawa et al. [56]. For this assay, 200 μL of supernatant was mixed with 0.1 M HCl with 1 mL of 50% TCA in 0.1 M HCl and 1 mL of 26 mM thiobarbituric acid. After vortex mixing, samples were held at 95 °C for 20 min. The samples were then centrifuged at 960× *g* for 10 min, and the supernatants were read at 532 nm against the control. The concentration of MDA was presented as nmol/mg protein.

#### 2.5.7. Protein Concentration

The protein content was estimated through Bradford’s dye-binding assay. 0.8 mL of Roti^®^ Quant reagent (Carl Roth, Germany) was mixed with up to 200 μL of the sample, and the change in absorbance at 595 nm was measured using a DU^®^ 740 Life Science spectrophotometer (Beckman Coulter, Brea, CA, USA). A calibration curve in the range of 2 to 20 μg was constructed using bovine serum albumin (BSA). Protein concentration was expressed as μg BSA/μL of clear homogenate [57].

### 2.6. Statistical Analysis

All results are expressed as mean ± standard error of the mean (S.E.M) and were analyzed by GraphPad Prism 8.0 software (GraphPad Software, Inc., San Diego, CA, USA). The normality of data distribution was evaluated using Shapiro-Wilk-Test. Datasets with multiple comparisons were evaluated using one-way ANOVA followed by Tukey’s *post hoc* test. *p* < 0.05 was considered to indicate a statistically significant difference. In the preference experiments within the Y-maze and NOR tests, one sample *t*-test was used for comparison of the preference to chance level (50%). The Pearson correlation coefficient (r) was used to estimate the correlation between the behavioral scores, enzymatic activities, and lipid peroxidation.

## 3. Results and Discussion

### 3.1. The Chemical Composition of the Thymus vulgaris Essential Oil

From a chemical point of view, TEO is generally characterized by a large amount of monoterpenes (both hydrocarbons and oxygenated) reaching almost 90% of the whole oil. The two main phenolic monoterpenes, thymol, and carvacrol occur more frequently. These are always accompanied by *p*-cymene and γ-terpinene being strictly biogenetically correlated with thymol and carvacrol [58,59]. *Thymus* is probably the most taxonomically complex genus of the *Lamiaceae* family. Several studies confirming the presence of a significant intraspecific chemical diversity in which the two most common chemotypes, thymol, and carvacrol, are preponderant [60], followed by less common non-phenolic chemotypes [61]. In particular, the sample subject of this study has a chemical profile quite typical of *Thymus vulgaris*, thymol chemotype. Gas chromatography analysis allowed the identification of more than 70 components covering more than 98% of the total oil composition. Table 1 shows the detail of the chemical composition, listing only the 41 components with the percentage >0.05. The most represented class is that of oxygenated monoterpenes (59.95%), followed by hydrocarbon monoterpenes (29.45%), the sum of which reaches 89%. Sesquiterpenes and other components are below 10%. The main compound is thymol (42.10%), followed by *p*-cymene (19.20%) and the sesquiterpene β-caryophyllene (6.40%). Carvacrol is below 3%.

Our findings are endorsed by Tardugno et al. [62], who reported that the main constituents of TEO were thymol (35.84–41.15%), *p*-cymene (17.50–21.73%), γ-terpinene (15.06–18.42%), linalool (2.55–5.37%), and carvacrol (1.45–1.70%). Csikós et al. [63] reported that thymol (46.3%) and *p*-cymene (22.1%) were identified as the main components of TEO. Rinaldi et al. [64] showed the presence of thymol as the most abundant (44.4%) followed by O-cymene (18.2%) and linalool (7.5%) in the chemical composition of TEO. Based on these results, our essential oil shows a chemical composition proportionate to those mentioned by other authors who assume its memory-enhancing and antioxidant function.

### 3.2. Impact on Anxiety-Like Behavior in NTT and on Spatial Memory in Y-Maze and NOR Tests

**Figure 2:**
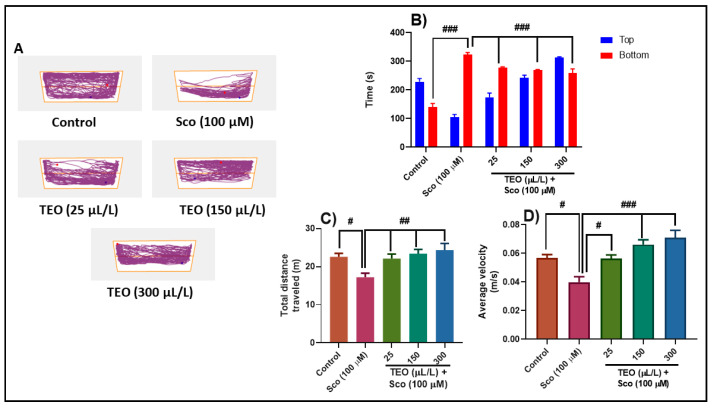
*Thymus vulgaris* essential oil (TEO: 25, 150, and 300 μL/L) improved locomotion pattern and reduced anxiety in the novel tank diving (NTT) test. (**A**) Representative locomotion tracking pattern of the control, scopolamine (Sco: 100 μM), and TEO (25, 150, and 300 μL/L) treated groups. (**B**) Represent the time spent in the top/bottom zone by zebrafish in the tank in different groups. (**C**) Represent the total distance travelled by zebrafish in the tank in different groups. (**D**) Represent the average velocity of the zebrafish in different groups. Values are means ± S.E.M. (*n* = 10). For Tukey’s *post hoc* analyses: (**B**) Control vs. Sco (100 μM): ### *p* < 0.0001, Sco (100 μM) vs. TEO (25 μL/L): ### *p* < 0.0001, Sco (100 μM) vs. TEO (150 μL/L): ### *p* < 0.0001, and Sco (100 μM) vs. TEO (300 μL/L): ### *p* < 0.0001; (**C**) Control vs. Sco (100 μM): # *p* < 0.01, Sco (100 μM) vs. TEO (25 μL/L): ## *p* < 0.001, Sco (100 μM) vs. TEO (150 μL/L): ## *p* < 0.001, and Sco (100 μM) vs. TEO (300 μL/L): ## *p* < 0.001; (**D**) Control vs. Sco (100 μM): # *p* < 0.01, Sco (100 μM) vs. TEO (25 μL/L): # *p* < 0.01, Sco (100 μM) vs. TEO (150 μL/L): ### *p* < 0.0001, and Sco (100 μM) vs. TEO (300 μL/L): ### *p* < 0.0001.

The NTT assesses the anxiety reaction evoked by novelty. Representative locomotion tracking patterns (Figure 2A) shows the variations in swimming traces within the NTT test between the top and the bottom areas. Sco-treated groups displayed a bottom zone preference which indicated high levels of anxiety. In the NTT test, one-way ANOVA revealed significant effect of the treatment on the time spent in the top/bottom zone of the tank (F (4.90) = 32.65, *p* < 0.0001) (Figure 2B), on the total distance traveled (F (4.45) = 4.87, *p* < 0.001) (Figure 2C), and on the average velocity (F (4.45) = 10.99, *p* < 0.0001) (Figure 2D). Sco treatment significantly increased the time spent in the bottom zone of the tank (*p* < 0.0001) (Figure 2B) compared with the control group. By decreasing the total distance traveled in the tank (*p* < 0.01) (Figure 2C) and the average velocity (*p* < 0.01) (Figure 2D), Sco treatment induced a hypolocomotor effect compared with the control group. The anxiolytic-like effect of the TEO treatment was noticed by decreasing the time spent in the bottom zone of the tank (*p* < 0.0001) (Figure 2B) as compared with the Sco-alone treated animals. Moreover, treatment with TEO prevents the hypolocomotor effect of Sco, in a dose-dependent manner, as evidenced through increasing the total distance traveled (*p* < 0.001) (Figure 2C) and the average velocity (*p* < 0.01 for the 25 μL/L and *p* < 0.0001 for the 150 μL/L and 300 μL/L) (Figure 2D) as compared with Sco-alone treated fish.

**Figure 3:**
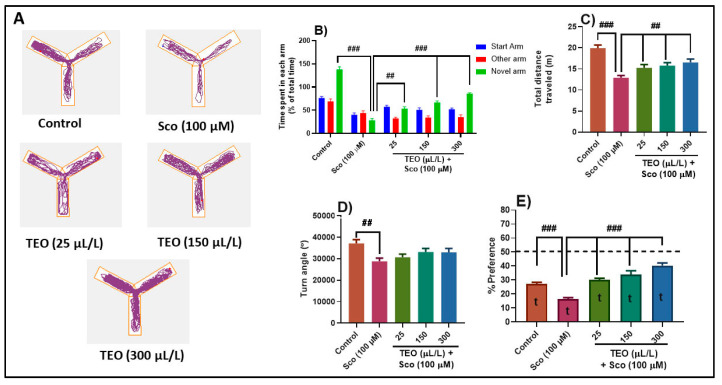
*Thymus vulgaris* essential oil (TEO: 25, 150, and 300 μL/L) improved locomotion pattern and memory in the Y-maze test. (**A**) Representative locomotion tracking pattern of the control, scopolamine (Sco: 100 μM), and TEO (25, 150, and 300 μL/L) treated groups. (**B**) Represent time spent in each arm (start, novel and novel arm) in different groups. (**C**) Represent the total distance traveled by zebrafish in the tank in different groups. (**D**) Represent the turn angle of zebrafish in the tank in different groups. Values are means ± S.E.M. (*n* = 10). For Tukey’s *post hoc* analyses: (**B**) Control vs. Sco (100 µM): ### *p* < 0.0001, Sco vs. TEO (25 µL/L): ## *p* < 0.001, Sco vs. TEO (150 µL/L): ### *p* < 0.0001 and Sco vs. TEO (300 µL/L): ### *p* < 0.0001; (**C**) Control vs. Sco (100 µM): ### *p* < 0.0001, Sco vs. TEO (25 µL/L): ## *p* < 0.001, Sco vs. TEO (150 µL/L): ## *p* < 0.001 and Sco vs. TEO (300 µL/L): ## *p* < 0.001; (**D**) Control vs. Sco (100 µM): ## *p* < 0.001; (**E**) Control vs. Sco (100 µM): ### *p* < 0.0001, Sco vs. TEO (25 µL/L): ### *p* < 0.0001, Sco vs. TEO (150 µL/L): ### *p* < 0.0001 and Sco vs. TEO (300 µL/L): ### *p* < 0.0001.

The typical locomotion tracking pattern (Figure 3A) illustrates the differences in swimming traces among the Y-maze arms. Sco-exposed zebrafish explored the novel arm less, suggesting deficits in the response to novelty. In the Y-maze test, one-way ANOVA revealed significant effect of the treatment on the time spent in each arm (F (8.90) = 19.60, *p* < 0.0001) (Figure 3B), on the total distance traveled (F (4.45) = 12.47, *p* < 0.0001) (Figure 3C), the turn angle (F (4.45) = 3.53, *p* < 0.01) (Figure 3D) and the preference percentages (F (4.45) = 25.36, *p* < 0.0001) (Figure 3E). Sco administration decreased the time spent in the novel arm (*p* < 0.0001) (Figure 3B) as compared with the control group, suggesting memory impairment. Besides, Sco treatment-induced hypolocomotion as evidenced by a decrease of the total distance traveled (*p* < 0.0001) (Figure 3C) and the turn angle (*p* < 0.001) (Figure 3D), as compared with the control group. TEO significantly improved memory deficits as evidenced by an increase of the time spent in the novel arm, in a dose-dependent manner (*p* < 0.001 for 25 μL/L and *p* < 0.0001 for 150 μL/L and 300 μL/L) (Figure 3B), as compared to Sco-alone treated fish. Moreover, TEO prevented Sco-induced hypolocomotion by significantly increased the total distance traveled in the tank (*p* < 0.001) (Figure 3C). Control group showed preferences for the novel arm in the Y-maze test, while Sco-treated zebrafish displayed less percentage preferences for the novel arm (*p* < 0.0001) (Figure 3E) as compared to control group, suggesting an impaired response to novelty. Besides, administration of TEO (25, 150, and 300 μL/L) in the Sco-treated animals, significantly improved the preference percentages (*p* < 0.0001 for 25, 150, and 300 μL/L) suggesting memory-enhancing profile (Figure 3E). Furtheremore, the preference percentage in control (t = 21.21, *p* < 0.0001), Sco (t = 9.13, *p* < 0.0001), TEO (25 µL/L) + Sco (t = 21.86, *p* < 0.0001), TEO (150 µL/L) + Sco (t = 11.98, *p* < 0.0001), and TEO (300 µL/L) + Sco (t = 25.59, *p* < 0.0001) groups were statistically different from the chance level (50%) (Figure 3E).

**Figure 4:**
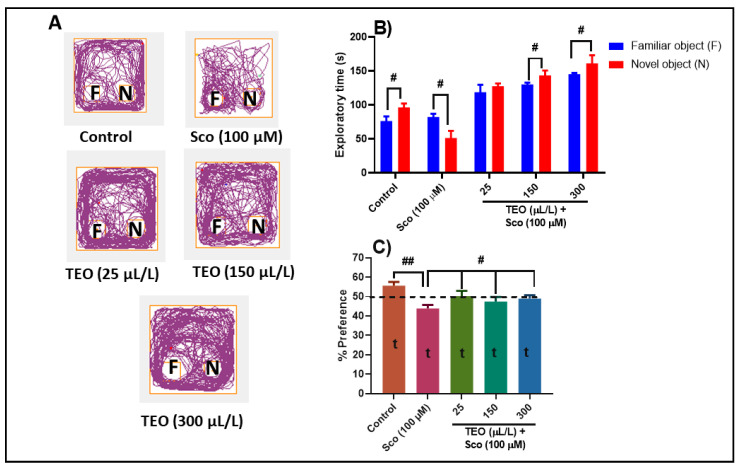
*Thymus vulgaris* essential oil (TEO: 25, 150, and 300 μL/L) improved memory in the novel object recognition (NOR) test. (**A**) Representative locomotion tracking pattern of the control, scopolamine (Sco: 100 μM), and TEO (25, 150, and 300 μL/L) treated groups; (**B**) Represent the exploratory time in different groups; (**C**) Represent the percentages of preference in different groups. Values are means ± S.E.M. (*n* = 10). For Tukey’s *post hoc* analyses: (**B**) # *p* < 0.01; (**C**) Control vs. Sco (100 µM): ## *p* < 0.001, Sco vs. TEO (25 µL/L): # *p* < 0.01, Sco vs. TEO (150 µL/L): # *p* < 0.01, and Sco vs. TEO (300 µL/L): # *p* < 0.01.

Representative locomotion tracking pattern (Figure 4A) illustrates the differences in the exploration of the familiar object (F) and the novel object (N) within the NOR. In the NOR test, one-way ANOVA revealed significant effect of treatment on the exploratory time (F (4.90) = 31.38, *p* < 0.0001) (Figure 4B) and the preference percentages (F (4.45) = 3.99, *p* < 0.001) (Figure 4C). Control group displayed a high preference to explore the N (*p* < 0.01), whereas Sco-treated group exhibited a high preference (*p* < 0.01) to explore F rather than to explore N, indicating memory impairment (Figure 4B) The Sco-treated zebrafish subjected to either 150 µL/L and 300 µL/L of TEO showed a significantly higher preference for the N instead of F, suggesting memory improvement profile (Figure 4B). Animals treated with Sco showed fewer percentages of preference (*p* < 0.001) (Figure 4C), as compared with the control group, whereas administration of TEO in the Sco-treated fish improved the percent of preferences for the N (*p* < 0.01 for the 25, 150, and 300 μL/L), suggesting a memory-enhancing profile. Furheremore, the preference percentage in control (t = 29.15, *p* < 0.0001), Sco (t = 23.22, *p* < 0.0001), TEO (25 µL/L) + Sco (t = 18.42, *p* < 0.0001), TEO (150 µL/L) + Sco (t = 19.64, *p* < 0.0001), and TEO (300 µL/L) + Sco (t = 27.90, *p* < 0.0001) groups were statistically different from the chance level (50%) (Figure 4C).

Additionally, in the control groups exposed to 25, 150, and 300 μL/L doses of TEO, a significant effect on the time spent in top/bottom zone and total distance traveled by zebrafish in the NTT test, along with a significant increase of the time spent in the novel arm of the Y-maze test and the exploratory time of the novel object in the NOR test were noticed (Figure S1).

Our results are in line with those recently published by other authors who reported an improvement of cognitive function following the administration of *Thymus vulgaris.* Akan et al. [65] demonstrated the positive effects of *Thymus vulgaris* L. and *Thymbra spicata* L. against diabetes mellitus-induced neuropathy and cognitive impairment as assessed by Morris water maze in rats. The authors attributed the observed effects to the presence in high amounts in the essential oil composition of some components such as thymol, carvacrol, 8-terpinene, *p*-cymene, and *α*-pinene. Rabiei et al. [37] suggested that the anti-amnesic effect of the *Thymus vulgaris* extract on Sco-induced memory deficits in rats by using Morris water maze and passive avoidance tests may be related to its antioxidants activity or the mediation of the cholinergic system activity. Again, the authors attributed these effects to carvacrol and thymol as evidenced in the chemical composition. Based on these results, TEO could be considered as an alternative tool for improving cognitive deficits in AD-dementia conditions.

### 3.3. Effects on AChE Activity

**Figure 5:**
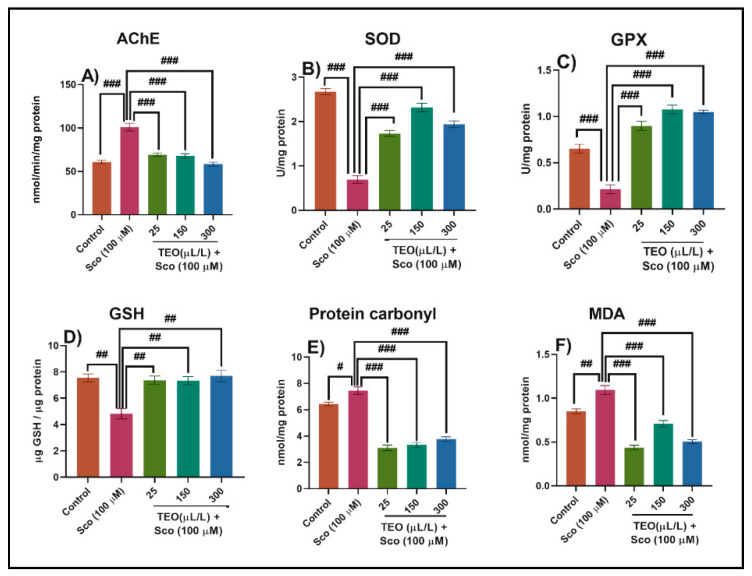
*Thymus vulgaris* essential oil (TEO: 25, 150, and 300 μL/L) exhibited an anti- acetylcholinesterase (AChE) effect and improved brain antioxidant status. The enzyme’s specific activities: (**A**) AChE; (**B**) superoxide dismutase (SOD); (**C**) glutathione peroxidase (GPX); (**D**) reduced glutathione (GSH); (**E**) protein carbonyl and (**F**) malondialdehyde (MDA) level. Values are means ± S.E.M. (*n* = 10). For Tukey’s *post hoc* analyses: (**A**) Control vs. Sco (100 µM): ### *p* < 0.0001, Sco vs. TEO (25 µL/L): ### *p* < 0.0001, Sco vs. TEO (150 µL/L): ### *p* < 0.0001 and Sco vs. TEO (300 µL/L): ### *p* < 0.0001; (**B**) Control vs. Sco (100 µM): ### *p* < 0.0001, Sco vs. TEO (25 µL/L): ### *p* < 0.0001, Sco vs. TEO (150 µL/L): ### *p* < 0.0001 and Sco vs. TEO (300 µL/L): ### *p* < 0.0001; (**C**) Control vs. Sco (100 µM): ### *p* < 0.0001, Sco vs. TEO (25 µL/L): ### *p* < 0.0001, Sco vs. TEO (150 µL/L): ### *p* < 0.0001 and Sco vs. TEO (300 µL/L): ### *p* < 0.0001; (**D**) Control vs. Sco (100 µM): ## *p* < 0.001, Sco vs. TEO (25 µL/L): ## *p* < 0.001, Sco vs. TEO (150 µL/L): ## *p* < 0.001 and Sco vs. TEO (300 µL/L): ## *p* < 0.001; (**E**) Control vs. Sco (100 µM): # *p* < 0.01, Sco vs. TEO (25 µL/L): ### *p* < 0.0001, Sco vs. TEO (150 µL/L): ### *p* < 0.0001 and Sco vs. TEO (300 µL/L): ### *p* < 0.0001; and (**F**) Control vs. Sco (100 µM): ## *p* < 0.001, Sco vs. TEO (25 µL/L): ### *p* < 0.0001, Sco vs. TEO (150 µL/L): ### *p* < 0.0001 and Sco vs. TEO (300 µL/L): ### *p* < 0.0001.

The levels of biochemical markers linked to cholinergic function and oxidative stress were examined to elucidate the underlying mechanism of TEO’s memory enhancement behavior in Sco-treated fish. Dysfunction and loss of cholinergic neurons in the basal forebrain have been reported to be among the earliest pathological events that occur in AD pathogenesis and are followed by a decline in the frequency of the choline acetyltransferase (ChAT) and AChE and acetylcholine (ACh) levels in the brains of people with AD [66]. The change in AChE activity is related to AD progression [67]. The AChE specific activity was significantly increased in the Sco-treated zebrafish compared with the control group (*p* < 0.0001) (Figure 5A). However, the TEO treatment significantly decreased (*p* < 0.0001) (Figure 5A) the AChE specific activity on all doses, and this parallel with the improvement of memory parameters, as evidenced in the behavioral approaches (NTT, Y-maze, and NOR tests). Dandlen et al. [68] demonstrated the AChE inhibition activity of portuguese *Thymus* species essential oils. Among the *Thymus* essential oils examined, practically all carvacrol, borneol, or 1,8-cineole rich oils have an inhibitory effect on AChE, indicating that these compounds are important for their bioactivity. Aazza et al. [69], demonstrated that TEO exhibited anti-AChE activity mainly due to the presence of the phenolic monoterpenes thymol and carvacrol. Owokotomo et al. [70] reported AChE inhibition activity of the essential oil from *Thymus vulgaris.* Kindl et al. [71] highlighted the anticholinesterase potential of six *Thymus* species. Our data indicate that TEO possesses cholinesterase inhibitory potential, related to the improvement of cognitive dysfunction in Sco-induced amnesic zebrafish.

### 3.4. Effects on the Brain Oxidative Status

We investigated SOD and GPX activities and the total content of reduced GSH, protein carbonyl, and MDA levels in the brain tissue to determine whether TEO inhibits oxidative damage induced by Sco. Sco treatment resulted in significant decrease in the specific activities of the antioxidants enzymes—SOD (*p* < 0.0001) (Figure 5B), GPX (*p* < 0.0001) (Figure 5C), the total content of reduced GSH (*p* < 0.001) (Figure 5D), along with increased levels of protein carbonyl (*p* < 0.01) (Figure 5E) and lipid peroxidation (MDA) (*p* < 0.001) (Figure 5F) when compared with the control group. Besides, TEO co-administration significantly ameliorated Sco-induced oxidative stress by enhancing the activity of the antioxidant enzymes and suppressing the levels of protein carbonyl and lipid peroxidation relative to Sco-treated animals.

Oxidative stress is associated with memory impairment in AD [72]. While ROS and free radicals such as superoxide (O_2_^−^) and hydrogen peroxide (H_2_O_2_) are produced during normal metabolism, an imbalance between the ROS production and antioxidant leads to oxidative stress in the body [73]. The body has several antioxidant enzymes, such as SOD and GPX to protect it from oxidative stress [73]. SOD acts as the first-line defense against oxidative stress, catalyzes O_2_^−^ to oxygen (O_2_) and H_2_O_2_, and then H_2_O_2_ is transformed into H_2_O by catalase [74]. Furthermore, GPX reduces both H_2_O_2_ and hydroperoxides by expending glutathione (GSH) [75]. In particular, SOD and GPX play an important role in protecting against oxidative stress in the brain [75,76]. MDA is one of the most significant aldehydes formed after lipid hydroperoxide breakdown. Hence the involvement of free radical damage in pathologies associated with oxidative stress is considered a good biomarker [77]. Furthermore, many studies reported that Sco-induced memory loss is related to increased brain oxidative stress [78,79]. Sco also significantly increases AChE and MDA levels in the cortex and hippocampus [80]. TEO significantly restored the antioxidant status in the zebrafish brain, as evidenced by a significant increase of SOD and GPX activities along with the decrease of the protein carbonyl and MDA levels. Our results are supported by literature concerning the antioxidant effects of the *Thymus*. By inhibiting lipid peroxidation and activation of the Keap1/Nrf2 pathway in zebrafish, *Thymus quinquecostatus* Celak. oils have provided protection against oxidative stress in zebrafish, as demonstrated by He et al. [81]. TEO treatment effectively restored the antioxidant defense mechanism by increasing the antioxidant levels of activity in the brain.

**Figure 6:**
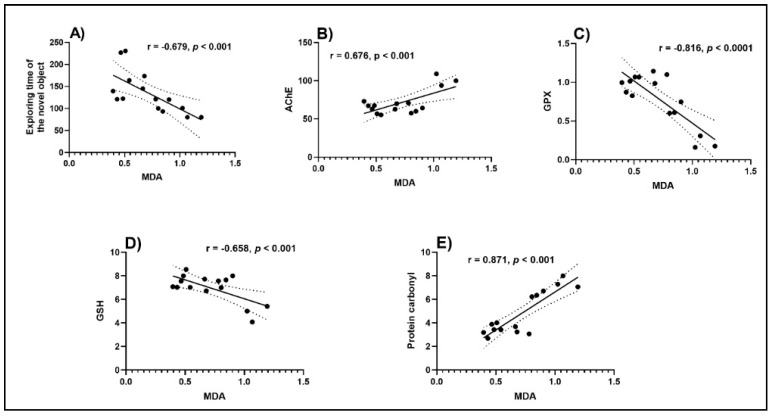
Correlation analyses between behavioral and biochemical parameters (Pearson’s correlation, *n* = 10). Data expressed are exploring time of the novel object (s), AChE (nmol/min/mg protein), GPX (U/mg protein), GSH (μg GSH/ μg protein), protein carbonyl (nmol/mg protein), and MDA (nmol/mg protein). (**A**) Exploring time of the novel object vs. MDA (*n* = 10, *r* = −0.679, *p* < 0.001); (**B**) AChE vs. MDA (*n* = 10, *r* = 0.676, *p* < 0.001); (**C**) GPX vs. MDA (*n* = 10, *r* = −0.816, *p* < 0.0001); (**D**) GSH vs. MDA (*n* = 10, *r* = −0.658, *p* < 0.001) and (**E**) protein carbonyl vs. MDA (*n* = 10, *r* = 0.871, *p* < 0.001).

Pearson correlation coefficient (*r*) was used to evaluate the relationship between cognition, antioxidant enzymes, and lipid peroxidation (Figure 6). A high negative correlation between the exploring time of the novel object vs. MDA (*n* = 10, *r* = −0.679, *p* < 0.001) (Figure 6A) was noticed. The negative value of the *r* indicates that the improvement of behavioral scores in NOR is well correlated with a low level of MDA, a marker of lipid peroxidation. Additionally, high negative correlations were evidenced by linear regression between GPX vs. MDA (*n* = 10, *r* = −0.816, *p* < 0.0001) (Figure 6C) and GSH vs. MDA (*n* = 10, *r* = −0.658, *p* < 0.001) (Figure 6D). However, a positive significant correlation between AChE vs. MDA (*n* = 10, *r* = 0.676, *p* < 0.001) (Figure 6B) and protein carbonyl vs. MDA (*n* = 10, *r* = 0.871, *p* < 0.001) (Figure 6E) was noticed when linear regression was calculated. In this case, the positive values of the *r* indicate that decreasing of ACh activity and protein carbonyl level is well correlated with decreasing of the MDA level. Kindl et al. [71] also demonstrated a relationship between the antioxidant and anticholinesterase potential of the *Thymus* species. By using the determination of the *r*, we have shown that improvement of the memory performance in Sco-treated rats is linked to increased antioxidant enzyme activity along with a decreased lipid peroxidation level, supporting the neuroprotective profile of TEO.

## 4. Conclusions

Considering a very wide range of evidence supporting oxidative stress involvement in neurodegenerative pathologies as well as the fact that AChE inhibition is up to now the most effective therapeutic approach to dementia, this study aimed to evaluate the neuroprotective potential of the TEO by investigating its cognitive-enhancing, anti-AChE and antioxidant activities in Sco zebrafish model. Our study suggested that the cognitive-protecting activities of TEO on Sco-induced memory impairment might result from its effect on improving the cholinergic nervous system and antioxidative stress. To sum up, TEO might be a promising candidate for the treatment of cognitive dysfunction.

## Figures and Tables

**Table 1:** Chemical composition of commercial *Thymus vulgaris* essential oil.

# ^a^	RI Lit ^b^	RI Exp ^c^	Class/Compound ^d^	% ^e^
			**Monoterpene hydrocarbons**	**29.45**
2	927	923	Tricyclene	0.07
3	930	927	α-Thujene	0.30
4	939	934	α-Pinene	1.52
5	954	949	Camphene	1.73
7	991	988	β-Myrcene	1.63
8	1003	1001	α-Phellandrene	0.12
9	1017	1015	α-Terpinene	0.89
10	1025	1025	*p*-Cymene	19.20
11	1029	1028	Limonene	0.48
13	1060	1058	γ-Terpinene	3.43
16	1089	1085	Terpinolene	0.08
			**Oxygenated monoterpenes**	**59.95**
12	1031	1031	1,8-Cineole	1.44
14	1070	1067	*cis* Sabinene hydrate	0.11
15	1073	1072	*trans* Linalool oxide	0.06
17	1097	1096	Linalool	5.67
18	1146	1141	Camphor	1.05
19	1153	1149	Menthone	1.08
20	1169	1163	Borneol	1.79
21	1172	1169	Menthol	0.48
22	1177	1174	Terpinen-4-ol	1.97
23	1183	1186	<iso>-Menthol	0.10
24	1189	1190	α-Terpineol	0.11
25	1235	1227	Thymol methyl ether	0.37
26	1245	1237	Carvacrol methyl ether	0.51
27	1238	1245	Neral	0.05
28	1242	1249	Carvone	0.31
29	1291	1281	*p*-Cymen-7-ol	0.05
30	1290	1295	Thymol	42.10
31	1299	1300	Carvacrol	2.70
			**Sesquiterpenes**	**7.93**
32	1351	1344	α-Cubebene	0.05
34	1377	1369	α-Copaene	0.15
35	1419	1411	β-Caryophyllene	6.40
36	1466	1450	<9-epi-(*E*)>-Caryophyllene	0.05
37	1485	1481	Germacrene D	0.08
38	1514	1500	γ-Cadinene	0.06
39	1523	1506	δ-Cadinene	0.23
40	1583	1568	Caryophyllene oxide	0.80
41	1670	1652	<14-Hydroxy-9-epi-(*E*)>-Caryophyllene	0.11
			**Others**	**1.32**
1	855	858	3-Hexen-1-ol	0.23
6	979	978	1-Octan-3-ol	0.87
33	1359	1350	Eugenol	0.22

^a^ The numbering refers to elution order; ^b^ Literature retention index (RI); ^c^ Retention index (RI) relative to a standard mixture of *n*-alkanes on SPB^TM^-5 column; ^d^ Identified compounds (<0.05% have not been reported); ^e^ Relative peak area percent represent averages of 3 determinations.

## Supplementary Materials

The following are available online at https://www.mdpi.com/2076-3921/9/11/1083/s1, Figure S1: The effects of *Thymus vulgaris* essential oil (TEO: 25, 150, and 300 μL/L) on the time spent in top/bottom zone and total distance traveled by zebrafish in the NTT test, the time spent in the novel arm of the Y-maze test and the exploratory time of the novel object in the NOR test in the control groups.
