# Supplementary material for: Thymus vulgaris Essential Oil Protects Zebrafish against Cognitive Dysfunction by Regulating Cholinergic and Antioxidants Systems
Source: Antioxidants (Basel). 2020 Nov 4;9(11):1083. doi: 10.3390/antiox9111083 (PMC7694219; doi:10.3390/antiox9111083)
Supplement: Supplementary file 1 [file antioxidants-09-01083-s001.pdf]

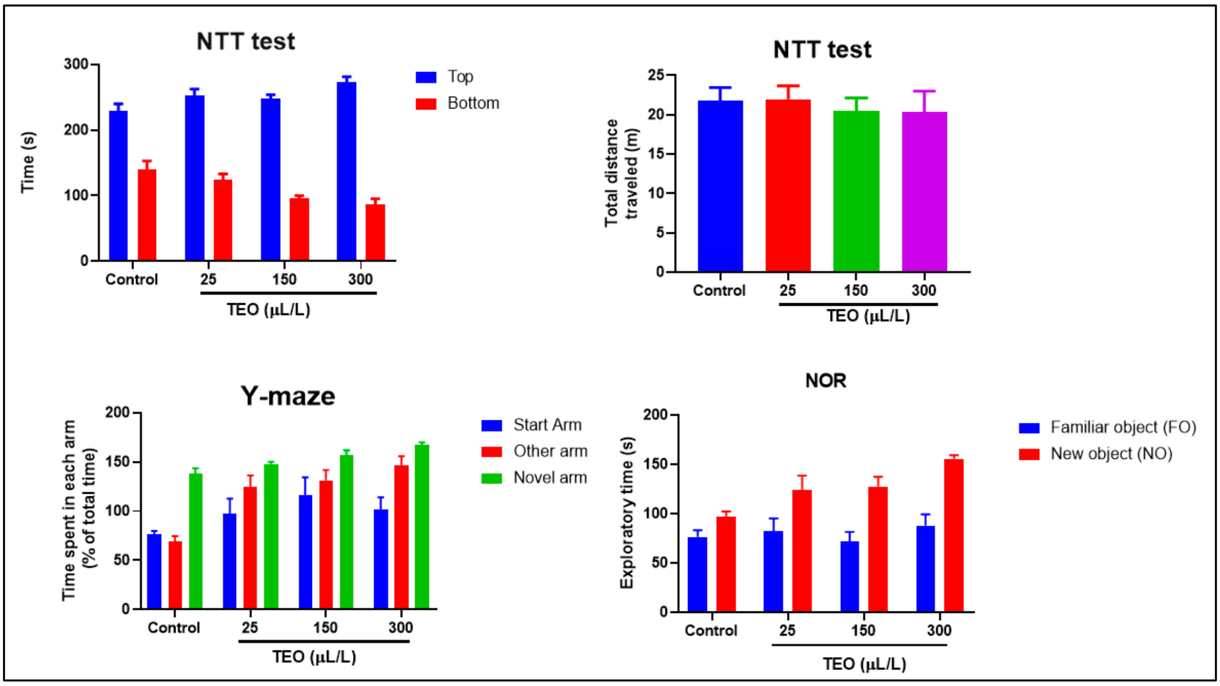

Figure S1. Th effects of *Thymus vulgaris* essential oil (TEO: 25, 150, and 300 µL/L) on the time spent in top/bottom zone and total distance traveled by zebrafish in the NTT test, the time spent in the novel arm of the Y-maze test and the exploratory time of the novel object in the NOR test in the control groups.
